# A case-only study of gene-environment interaction between genetic susceptibility variants in *NOD2 *and cigarette smoking in Crohn's disease aetiology

**DOI:** 10.1186/1471-2350-13-14

**Published:** 2012-03-14

**Authors:** Katherine L Helbig, Michael Nothnagel, Jochen Hampe, Tobias Balschun, Susanna Nikolaus, Stefan Schreiber, Andre Franke, Ute Nöthlings

**Affiliations:** 1 Institute for Experimental Medicine, Section of Epidemiology, Christian-Albrechts-University of Kiel, Arnold-Heller-Straβe 3, Haus 3, 24105 Kiel, Germany; 2 Institute of Medical Informatics and Statistics, Christian-Albrechts-University of Kiel, Kiel, Germany; 3 Department of General Internal Medicine, University Hospital Schleswig-Holstein, Christian-Albrechts-University of Kiel, Kiel, Germany; 4 Institute for Clinical Molecular Biology, Christian-Albrechts-University of Kiel, Kiel, Germany; 5 Biobank popgen, University Hospital Schleswig-Holstein, Christian-Albrechts-University of Kiel, Kiel, Germany

**Keywords:** Gene-environment interaction, Case-only, Crohn's disease, *NOD2/CARD15*, Cigarette smoking

## Abstract

**Background:**

Genetic variation in *NOD2 *and cigarette smoking are well-established risk factors for the development of Crohn's disease (CD). However, little is known about a potential interaction between these risk factors. We investigated gene-environment interactions between CD-associated *NOD2 *alleles and cigarette smoking in a large sample of patients with CD.

**Methods:**

Three previously reported CD-associated variants in *NOD2 *(R702W, G908R, 1007fs) were genotyped in 1636 patients with CD continuously recruited between 1995 and 2010 based on physician referral. Data on history of smoking behaviour was obtained for all participants through a written questionnaire. Using a case-only design, we performed logistic regression analyses to investigate statistical interactions between *NOD2 *risk alleles and smoking status.

**Results:**

We detected a significant negative interaction between carriership of at least one of the *NOD2 *risk alleles and history of ever having smoked (OR = 0.71; *p *= 0.005) as well as smoking at the time of CD diagnosis (OR = 0.68; *p *= 0.005). Subsequent separate analyses of the three variants revealed a significant negative interaction between the 1007fs variant and history of ever having smoked (OR = 0.64; *p *= 9 × 10^-4^) and smoking at the time of CD diagnosis (OR = 0.53; *p *= 7 × 10^-5^).

**Conclusions:**

The observed significant negative gene-environment interaction suggests that the risk increase for CD conferred simultaneously by cigarette smoking and the 1007fs *NOD2 *polymorphism is smaller than expected and may point to a biological interaction. Our findings warrant further investigation in epidemiological and functional studies to elucidate pathophysiology as well as to aid in the development of recommendations for disease prevention.

## Background

Crohn's disease (CD) is a common form of inflammatory bowel disease (IBD) characterised by chronic relapsing and remitting episodes of transmural inflammation of the gastrointestinal tract. High rates of CD are found in North America (including Canada), northern Europe, and the United Kingdom, with an estimated 47,000 new cases diagnosed each year in North America and 41,000 new cases diagnosed each year in Europe [1]. The disease burden of CD is high, often associated with complications such as intestinal strictures, fistulas, and granulomas, the need for surgical intervention, and extraintestinal manifestations [2]. It is believed that immune tolerance to normal intestinal bacteria is disturbed in genetically susceptible individuals, leading to a pathogenic inflammatory response [3].

Evidence from twin and familial aggregation studies [4], as well as more recent evidence from genome-wide association studies [5] suggests that genetic factors play an important role in CD aetiology. Among the first and most consistently replicated genetic associations discovered in CD were three variants in *NOD2 *(nucleotide-binding oligomerisation domain containing 2; previously known as *CARD15*) [6,7], which represent the strongest genetic risk factors for CD identified to date [8]. NOD2, an intracellular sensor of bacteria-derived muramyl dipeptide, plays an essential role in regulating host response to intestinal bacteria [9,10]. The three main reported disease-associated polymorphisms (R702W, G908R, and 1007fs) all lie within or near a leucine rich repeat domain, and it is thought that these three CD-associated variants contribute to disease pathogenesis through interference with bacterial recognition [11,12]. Associations between CD and the three main susceptibility variants are well-established [11,13,14] with a recent meta-analysis revealing disease odds ratios (OR) under a dominant genetic model of 2.2 (95% CI 2.0-2.5) for the R702W polymorphism, 2.6 (95% CI 2.2-2.9) for G908R, and 3.8 (95% CI 3.4-4.3) for 1007fs [15]. When data for all three variants are combined, the disease OR associated with simple heterozygotes is 2.4 (95% CI 2.0-2.8) and for compound heterozygotes is 9.0 (95% CI 6.0-13.5) [15].

While the association of *NOD2 *variants with CD is confirmed, the relatively small effect size of disease-associated *NOD2 *alleles even among homozygotes [16] and the observation that *NOD2 *knock-out mice do not develop spontaneous intestinal inflammation [9] indicate that additional factors are necessary for disease pathogenesis. Several environmental factors such as appendectomy, dietary factors, domestic hygiene, oral contraceptive use, and childhood infections have been implicated in CD [17], but the most consistent epidemiological evidence points to a clear risk associated with cigarette smoking [18,19], which is the strongest environmental risk factor identified for CD to date [20]. Two meta-analyses reveal that current cigarette smokers are up to twice as likely to develop CD as non-smokers (OR = 1.8-2.0) [21,22]. Furthermore, cigarette smoking is known to compromise innate and adaptive immune responses, leading to increased susceptibility to microbial infections [23], supporting its potential role in the pathogenesis of CD.

It has been speculated that an interaction between genetic and environmental factors is necessary for the development of complex genetic disorders including CD [24]. Although gene-environment interactions have often been invoked in explaining the aetiology of CD [25], studies of these interactions in CD have thus far received little research attention. Given the established role of NOD2 in regulating innate immune responses and cigarette smoke's impact on innate immunity, it is plausible that cigarette smoking might modulate the functional consequences of CD-associated polymorphisms in *NOD2*.

In this study, we used a case-only design to investigate potential gene-environment interactions between cigarette smoking and susceptibility variants in *NOD2 *among patients with CD. The case-only study design quantifies gene-environment interaction using the interaction odds ratio. Using this measure, the observed effect of both variables combined is compared to the hypothetical effect size when the effect of both factors is multiplied. Accordingly, an interaction odds ratio of 1.0 suggests the absence of interaction, whereas an interaction OR significantly greater than 1.0 suggests positive (synergistic) interaction and an interaction OR significantly less than 1.0 suggests negative (antagonistic) interaction. We hypothesised that the interaction between disease-associated *NOD2 *alleles and history of smoking would deviate from multiplicativity with regard to risk increase for CD (i.e. interaction OR significantly greater than or less than 1.0), thus being potentially indicative of biological interaction. We further hypothesised that this deviation would be more pronounced among patients who were active smokers at the time of CD diagnosis. The current study represents the largest study to date to examine statistical gene-environment interactions in patients with CD and the first to employ a case-only design.

## Results

### Study sample characteristics

Table 1 provides demographic and risk factor characteristics for the 1636 participants in the analytic sample according to smoking status. Data regarding history of ever having smoked was available for all 1636 participants. Smoking status at time of diagnosis was available for 1283 (78%) participants. The study sample included various subsamples recruited at different time points. Information on smoking at diagnosis was missing for the majority of patients in a single subsample with a high frequency of family history (n = 450, recruited from 2002 to 2004, 68.5% with missing data). However, a sensitivity analysis excluding this subsample did not significantly alter the results (Additional file 1: Table S1) and therefore, this subsample was included in the final analytic sample. Using a control population from the popgen biobank as a comparison group, we confirmed smoking as a risk factor for Crohn's disease in our study population (OR (95% CI) 1.24 (1.04-1.47), *p *= 0.016 for ever having smoked). Genotype data is also summarised in Table 1. Genotype status of all three polymorphisms was available for all 1636 participants. The study sample was largely comprised of women (69%). Female participants were slightly more likely than male participants to have been smokers at the time of CD diagnosis (*p *= 0.02). Participants who had ever smoked were on average older at diagnosis (mean age ever smokers 27.4 years, never smokers 24.1 years; *p *< 0.001) and at study inclusion (mean age ever smokers 40.8 years, never smokers 36.3 years; *p *< 0.001) than those who had no history of smoking. Similarly, individuals who were smokers at the time of disease diagnosis were on average older at diagnosis (mean age smokers 27.3 years, non-smokers 26.0 years; *p *= 0.03) and study inclusion (mean age smokers 41.1 years, non-smokers 38.8 years; *p *< 0.001) than non-smokers at diagnosis. Carriers of at least one CD-associated *NOD2 *allele were diagnosed at a younger age (mean age at diagnosis 25.1 years) than participants who were wild type at all three loci (mean age at diagnosis 26.8 years; *p *< = 0.001; Additional file 1: Table S2). Analyses of each polymorphism separately indicated that the 1007fs allele was the only polymorphism associated with an earlier age at diagnosis (mean age at diagnosis for carriers 24.6 years; mean age at diagnosis for non-carriers 26.5 years; *p *= 0.001; Additional file 1: Table S2). Age at diagnosis was not associated with the R702W and G908R polymorphisms. Sex, age at study inclusion, and family history of IBD were not associated with genotype for any of the three risk variants (Additional file 1: Table S2). A positive family history of IBD was not associated with smoking status in probands (*p *= 0.08, Chi square analysis). Analyses comparing the study participants included and excluded from the final analyses revealed no significant differences with regard to sex, smoking status, age at study inclusion, or genotype at any of the three loci (Additional file 1: Table S3). Excluded participants were diagnosed at a slightly younger age than included participants (24.8 years vs. 26.8 years; *p *< 0.001; Additional file 1: Table S3).

**Table 1:** Distribution of demographic and risk factor characteristics for all participants in analytic sample by smoking status

	Ever Smokers		Never Smokers			Smokers at Diagnosis		Non-Smokers at Diagnosis			Total Sample	
					
Characteristic	n	%	n	%	*p*-value	n	%	n	%	*p*-value	n	%
**Total Participants n = 1636**	945	57.8%	691	42.2%		522	40.7%	761	59.3%		1636	100.0%

**Sex**					0.09					**0.02**		
Men	281	29.7%	233	33.7%		144	27.6%	258	33.9%		514	31.4%
Women	664	70.3%	458	66.3%		378	72.4%	503	66.1%		1122	68.6%

**Age at Inclusion--Mean** **(Standard Deviation)**	40.8	± 10.9	36.3	± 12.9	**2 × 10**^**-13**^	41.1	± 10.6	38.8	± 13.5	**6 × 10**^**-4**^	38.9	± 12.0

**Age at Diagnosis--Mean** **(Standard Deviation)**	27.4	± 10.1	24.1	± 10.1	**7 × 10**^**-11**^	27.3	± 9.5	26.0	± 11.4	**0.03**	26	± 10.3

**Family History**					0.17					0.43		
Yes	229	24.2%	196	28.4%		75	14.4%	128	16.8%		425	26.0%
No	463	49.0%	320	46.3%		295	56.5%	408	53.6%		783	47.9%
Unknown	253	26.8%	175	25.3%		152	29.1%	225	29.6%		428	26.2%

**Combined Allele Analysis**					**1 × 10**^**-4**^					**0.001**		
Wild Type	549	58.1%	336	48.6%		311	59.6%	384	50.5%		885	54.1%
Carrier of ≥ 1 risk allele	396	41.9%	355	51.4%		211	40.4%	377	49.5%		751	45.9%

**R702W Polymorphism**					0.12					0.34		
Wild Type (C/C)	776	82.1%	540	78.1%		429	82.2%	608	79.9%		1316	80.4%
Heterozygote for risk allele (C/T)	153	16.2%	134	19.4%		85	16.3%	133	17.5%		287	17.5%
Homozygote for risk allele (T/T)	16	1.7%	17	2.5%		8	1.5%	20	2.6%		33	2.0%

**G908R Polymorphism**					0.93					0.12		
Wild Type (G/G)	858	90.8%	626	90.6%		475	91.0%	689	90.5%		1484	90.7%
Heterozygote for risk allele (G/C)	84	8.9%	62	9.0%		47	9.0%	66	8.7%		146	8.9%
Homozygote for risk allele (C/C)	3	0.3%	3	0.4%		0	0.0%	6	0.8%		6	0.4%

**1007fs Polymorphism**					**1 × 10**^**-4**^					**7 × 10**^**-6**^		
Wild Type (-/-)	735	77.8%	477	69.0%		420	80.5%	524	68.9%		1212	74.1%
Heterozygote for risk allele (-/C)	174	18.4%	166	24.0%		88	16.9%	187	24.6%		340	20.8%
Homozygote for risk allele (C/C)	36	3.8%	48	6.9%		14	2.7%	50	6.6%		84	5.1%

### Gene-environment interaction analysis

We observed a significant departure from multiplicativity between carriership of at least one of the three risk variants and history of ever having smoked (OR estimate of departure from multiplicativity adjusted for age at diagnosis, age at recruitment, sex, family history of IBD, and year of recruitment (AOR) = 0.71, 95% CI 0.56-0.90; Table 2) and history of smoking at the time of CD diagnosis (AOR = 0.68, 95% CI 0.51-0.89; Table 3). Upon analysis of individual polymorphisms, we observed a significant departure from multiplicativity only between the 1007fs risk allele and history of ever having smoked (AOR = 0.64, 95% CI 0.49-0.83; Table 2) and history of smoking at time of CD diagnosis (AOR = 0.53, 95% CI 0.39-0.73; Table 3). We found no evidence for interaction between the R702W and G908R risk alleles and history of ever smoking or smoking status at the time of diagnosis.

**Table 2:** Departure from multiplicativity between CD-associated *NOD2 *alleles and ever smoking status among patients with Crohn's disease

	Smoking status		Unadjusted			Adjusted*		
	
	Ever smoked	Never smoked	OR	95% CI	***p***-**value**	OR	95% CI	***p***-**value**
**Combined Allele Analysis**								
Wild type at all 3 loci	549 (58.1%)	336 (48.6%)	1.00	-	-	1.00	-	-
Carrier of ≥ 1 risk allele	396 (41.9%)	355 (51.4%)	0.68	0.56-0.83	1 × 10^-4^	**0.71**	**0.56-0.90**	**0.005**

**R702W Polymorphism**								
Wild type	776 (82.1%)	540 (78.1%)	1.00	-	-	1.00	-	-
Carrier of risk allele	169 (17.9%)	151 (21.9%)	0.78	0.61-1.00	0.04	0.80	0.59-1.07	0.14

**G908R Polymorphism**								
Wild type	858 (90.8%)	626 (90.6%)	1.00	-	-	1.00	-	-
Hetero- or homozygote	87 (9.2%)	65 (9.4%)	0.98	0.70-1.37	0.89	1.00	0.67-1.53	0.96

**1007fs Polymorphism**								
Wild type	735 (77.8%)	477 (69.0%)	1.00	-	-	1.00	-	-
Carrier of risk allele	210 (22.2%)	214 (31.0%)	0.64	0.51-0.80	7 × 10^-5^	**0.64**	**0.49-0.83**	**9 × 10**^**-4**^

* adjusted for sex, age at diagnosis, age at study inclusion, family history, and year of recruitment

**Table 3:** Departure from multiplicativity between CD-associated *NOD2 *alleles and smoking status at diagnosis among patients with Crohn's disease

	Smoking status at diagnosis		Unadjusted			Adjusted*		
	
	Smoker at diagnosis	Non-smoker at diagnosis	OR	95% CI	***p***-**value**	OR	95% CI	***p***-**value**
**Combined Allele Analysis**								
Wild type at all 3 loci	311 (59.6%)	384 (50.5%)	1.00	-	-	1.00	-	-
Carrier of ≥ 1 risk allele	211 (40.4%)	377 (49.5%)	0.69	0.55-0.87	0.001	**0.68**	**0.51-0.89**	**0.005**

**R702W Polymorphism**								
Wild type	429 (82.2%)	608 (79.9%)	1.00	-	-	1.00	-	-
Carrier of risk allele	93 (17.8%)	153 (20.1%)	0.86	0.65-1.14	0.30	0.82	0.57-1.16	0.27

**G908R Polymorphism**								
Wild type	475 (91.0%)	689 (90.5%)	1.00	-	-	1.00	-	-
Carrier of risk allele	47 (9.0%)	72 (9.5%)	0.94	0.64-1.39	0.78	0.93	0.57-1.49	0.76

**1007fs Polymorphism**								
Wild type	420 (80.5%)	524 (68.9%)	1.00	-	-	1.00	-	-
Carrier of risk allele	102 (19.5%)	237 (31.1%)	0.54	0.41-0.70	3 × 10^-6^	**0.53**	**0.39-0.73**	**7 × 10**^**-5**^

* adjusted for sex, age at diagnosis, age at study inclusion, family history, and year of recruitment

## Discussion

In an investigation of statistical interactions between cigarette smoking and CD-associated *NOD2 *polymorphisms, we found a significant negative interaction between the *NOD2 *1007fs polymorphism and cigarette smoking. We did not observe this interaction for the R702W or G908R polymorphisms. For both smoking variables analysed, i.e. history of ever having smoked and smoking status at the time of diagnosis, the observed interaction OR between smoking and the 1007fs allele was lower than expected. These results indicate that the increase in disease risk conferred by both cigarette smoking and the 1007fs allele is significantly lower than would be expected under a multiplicative model. Accordingly, the lower than expected disease risk in individuals who both carry the risk allele and smoke suggests a negative interaction between both factors.

It is possible that age of disease diagnosis and family history of IBD might confound these results, especially given the earlier age of disease diagnosis associated with the 1007fs variant. However, our final model was adjusted for age at diagnosis and family history, which controls for potential confounding effects. Furthermore, family history of IBD was not associated with smoking status in our cohort. This indicates that age at diagnosis and family history of IBD do not confound our results.

The strong negative interaction between cigarette smoking and the 1007fs variant is intriguing given the conflicting data from previous research. Statistical interactions between CD-associated *NOD2 *variants and smoking were previously investigated in two case-control studies, which failed to identify any statistical interactions between smoking status and *NOD2 *genotypes [13,26]. These studies, however, were limited by small sample sizes and lacked the statistical power to detect interactions of the size identified in our study. However, two small studies have suggested that smokers and non-smokers with CD have differences in their genetic backgrounds, including variants in *NOD2*, with the 1007fs variant found more commonly among non-smokers [27,28]. These previous findings are consistent with our results of a negative interaction between cigarette smoking and the 1007fs allele. Taken together, these data are suggestive that cigarette smoking modifies the disease risk associated with the 1007fs variant, and the increase in disease risk for smokers who carry this susceptibility allele compared to non-smokers who carry this allele is significantly less than expected.

To our knowledge the current study represents the largest analysis to date of statistical gene-environment interactions in CD aetiology and the first to employ a case-only design. Previous studies investigating gene-environment interaction in CD [13,26] did not employ the case-only design, which has been increasingly recognised over the last two decades as an attractive method for assessing the presence of statistical interaction [29-32]. The case-only design offers substantial increases in power as well as greater precision in estimating interactions when compared to traditional case-control designs [31].

Although our findings are promising, the current study does have limitations. The main limitation is that the case-only design only allows for the assessment of statistical interactions under a multiplicative model, which is considered by many epidemiologists to be less meaningful than an additive scale [33]. Furthermore, case-only designs are particularly sensitive to the assumption of independence of the genetic and environmental risk factors in the general population [34]. However, we observed no association between any of the CD-associated risk alleles and history of ever having smoked in a sample of 718 German population-based controls, which supports the assumption that these risk factors are independent. Additionally, a recent meta-analysis suggests that bias in case-only studies is rare, and no differences in OR estimates between traditional case-control and case-only studies of gene-environment interactions were observed [32]. Our study was further limited by the definition of smoking status. In the questionnaire administered to participants, no definition of active smoking was specified. Participants were asked only if they had ever smoked, which may have led to misclassification of the environmental variable among some participants. However, if the rates of misclassification of smoking status and genotypes are non-differential, that is if the classification of smoking status is independent of genotype classification, as one would expect, misclassification would push the interaction OR towards unity rather than generate an inflated estimate of interaction [33].

Despite these limitations, statistical interaction represents a powerful tool to point to potentially meaningful biological interaction. Evidence from functional studies [35] and an animal model of the 1007fs polymorphism [10] suggests that the protein product encoded by this allele exhibits gain of function properties, resulting in an impairment of innate immune tolerance towards commensal bacteria. The three CD-associated *NOD2 *polymorphisms furthermore have been shown to cause a failure to induce autophagy via ATG16L1, impairing autophagic wrapping and removal of invading bacteria [36]. It has also been observed that cigarette smoke extract delays *NOD2 *mRNA expression and impairs NOD2 functioning in intestinal epithelial cells [37]. It is reasonable to then speculate that the detrimental gain of function effects associated with the 1007fs associated protein may be modulated by the biological effects of cigarette smoking. The immunosuppressive properties of nicotine, one of the best studied components of cigarette smoke, are well-established [23] and have been shown to persist several weeks after exposure [38].

Our study indicates that smoking may modify the overall disease risk associated with established genetic risk variants. Therefore, it is plausible to suggest that similar gene-environment interactions may exist that influence the clinical manifestations and severity of CD. Indeed it has recently been demonstrated that cigarette smoking interacts with CD risk alleles, including *NOD2 *variants, to influence disease location in patients with CD [39]. Further research is necessary to elucidate the potential biological mechanisms behind such gene-environment interactions as well as to understand how behaviour modification could potentially benefit patients carrying disease-associated *NOD2 *variants.

## Conclusions

Our finding of a significant negative statistical interaction between cigarette smoking and the 1007fs *NOD2 *polymorphism in patients with Crohn's disease is intriguing and warrants further investigation in epidemiological and functional studies. Confirmation of a mechanistic interaction by which cigarette smoking modulates the risk associated with disease-associated *NOD2 *alleles may be important in not only better understanding the biological mechanisms underlying CD pathogenesis, but also in developing appropriate recommendations for individuals at high risk of developing CD.

## Methods

### Study design

Individuals with Crohn's disease (CD) were continuously recruited from 1995 to 2010 as part of ongoing studies for identifying genetic factors in inflammatory bowel diseases. Patients were ascertained from across Germany through physician referrals based on a positive diagnosis of CD. Established clinical, radiologic, and endoscopic criteria were required to confirm the diagnosis of CD, as previously described [40-42]. A peripheral venous blood sample was obtained from each participant at time of recruitment. Informed written consent was obtained from all study participants. The study protocol was approved by the ethics committee of the University Medical Hospital Schleswig-Holstein at the Christian-Albrechts-University of Kiel. All patients were recruited through the popgen biobank [43].

### Data collection

#### Study population

A total of 2430 German individuals with Crohn's disease were eligible for participation in the current study. Participants were excluded from analyses if genotype data for any of the three risk loci were unavailable or if they were non-proband members of multiplex families and thus related to other members of the study sample. Of the multiplex families, only the index patients were included in the final study sample. Participants were included in the analysis only if genotype data were available for all three CD-associated *NOD2 *polymorphisms. Genotype data were unavailable for 744 individuals, excluding them from further analysis. We excluded a further 50 participants, as they were non-proband members of multiplex families. After exclusion of related individuals and those with missing genotypes, a total of 1636 participants were available for analysis.

#### Smoking behaviour

Participants in the study were administered a written questionnaire to obtain information on demographic characteristics, disease specific information, general medical history, family history, and history of active cigarette smoking. Participants were asked to indicate if they had ever been active smokers and were assigned either a "never smoker" or "ever smoker" status according to responses given in the questionnaire. Participants were furthermore asked to indicate if they had been smokers at the time of CD diagnosis. Participants designated as "ever smokers" were further subdivided into either "smokers at time of diagnosis" or "non-smokers at time of diagnosis".

#### Genotyping

DNA was extracted from peripheral blood lymphocytes from each participant using standard methods. The R702W, G908R, and 1007fs polymorphisms were genotyped in all patients using TaqMan^® ^SNP genotyping assays as previously described [14].

### Statistical analysis

Demographic and risk factor variables for the included and excluded participants were compared using a χ^2 ^test for categorical variables and the independent samples t-test for continuous variables.

For the analysis of gene-environment interaction, we examined the effect of three established *NOD2 *risk polymorphisms (R702W, G908R, and 1007fs) and history of having ever smoked in patients with Crohn's disease. We employed a case-only study design, which uses data only from diseased individuals to estimate statistical gene-environment interactions under a multiplicative model, assuming that the genetic and environmental exposures are independent within the general population [29-31].

Genetic exposures were coded dichotomously (i.e. present or absent) for all analyses. In an initial combined allele analysis, participants who were carriers of at least one of the three risk variants were combined into a single group and compared to participants who were wild type at all three loci. In order to determine which polymorphisms were contributing to the signal detected in the combined variant analysis, we then performed subsequent analyses of each polymorphism separately. To investigate whether deviations from multiplicativity were stronger amongst participants who were smokers at the time of diagnosis, we performed a second set of analyses examining smoking status at the time of CD diagnosis.

To assess departure from multiplicativity between the presence of *NOD2 *polymorphisms and smoking status, we performed logistic regression analyses in R version 2.10.1 [44]. Presence of the *NOD2 *risk allele(s) of the polymorphism under consideration was designated as the response variable, while ever smoking status was used as the predictor variable in the first set of analyses and smoking status at the time of diagnosis was used as the predictor variable in the second set of analyses. Presence of gene-environment interaction was tested using a likelihood-ratio test in the regression model. Departure from multiplicativity between risk allele under consideration and smoking status was described by estimating odds ratios (ORs) for the interaction term in the regression model and respective 95% confidence intervals (CIs). Participants with wild type genotype and who were classified as never having smoked or as non-smokers at the time of CD diagnosis were used as the reference group for all respective analyses. All analyses were adjusted for the potential confounding effects of sex, age at diagnosis, age at recruitment, family history of IBD, and year of recruitment in a multiple logistic regression model.

Power calculations were performed for the combined allele analyses and for the analyses of each polymorphism separately using Quanto version 1.2.4 [45,46]. A case-only outcome design for gene-environment interactions under a dominant genetic model was used. Established estimates of disease prevalence [47], risk allele frequencies in the German general population [16], and smoking frequency in a German population-based control sample along with disease ORs associated with cigarette smoking and CD-associated *NOD2 *variants derived from meta-analyses [15,22] were used for power calculations. Power curves for each of the eight analyses performed are presented in Additional file 2: Figure S1 and Additional file 3: Figure S2. For the analyses of statistical interaction with history of ever having smoked, the current study had 80% statistical power to detect an interaction OR of 0.73 (combined allele analysis), 0.67 (R702W), 0.41 (G908R), and 0.70 (1007fs). Our study had 80% statistical power to detect an interaction OR of 0.70 (combined allele analysis), 0.64 (R702W), 0.35 (G908R), and 0.67 (1007fs) for the analyses of interaction with smoking status at the time of diagnosis.

Conservative Bonferroni correction for eight tests at an overall alpha level of 0.05 was applied to all analyses to correct for multiple comparisons.

## Authors' contributions

KLH designed the study, performed data analysis and interpretation, and wrote the manuscript. MN provided statistical and interpretational advice and support. JH, TB, and AF provided genotype and phenotype data for all participants. JH, SN, and SS were instrumental in participant recruitment, data collection, and phenotyping of participants. UN supervised and designed the study and wrote the manuscript. All authors reviewed, edited, and approved the final paper.

## Pre-publication history

The pre-publication history for this paper can be accessed here:

http://www.biomedcentral.com/1471-2350/13/14/prepub

## Supplementary Material

Additional file 1 **Table S1**. Sensitivity analysis comparing gene-environment interaction between the entire analytic sample (n = 1636) and a dataset excluding a subsample with incomplete smoking at diagnosis data (n = 1186); all analyses adjusted for sex, age at diagnosis, age at study inclusion, family history, and year of recruitment. **Table S2**. Comparison of sex, age at disease diagnosis, age at study inclusion, and family history of IBD for each of the CD-associated *NOD2 *risk variants among all participants included in the final analytic sample (n = 1636). **Table S3**. Comparison of demographic and risk variables between participants included and excluded from the final analytic study sample. Click here for file

Additional file 2 **Figure S1**. Estimates of statistical power to detect various interaction ORs for each of the four analyses examining history of ever having actively smoked. n = 1636. Click here for file

Additional file 3 **Figure S2**. Estimates of statistical power to detect various interaction ORs for each of the four analyses examining smoking status at time of CD diagnosis. Combined allele analysis n = 1283. Click here for file
