# Supplementary material for: A case-only study of gene-environment interaction between genetic susceptibility variants in NOD2 and cigarette smoking in Crohn's disease aetiology
Source: BMC Med Genet. 2012 Mar 14;13:14. doi: 10.1186/1471-2350-13-14 (PMC3314543; doi:10.1186/1471-2350-13-14)
Supplement: Additional file 1 — Table S1. Sensitivity analysis comparing gene-environment interaction between the entire analytic sample (n = 1636) and a dataset excluding a subsample with incomplete smoking at diagnosis data (n = 1186); all analyses adjusted for sex, age at diagnosis, age at study inclusion, family history, and year of recruitment. Table S2. Comparison of sex, age at disease diagnosis, age at study inclusion, and family history of IBD for each of the CD-associated NOD2 risk variants among all participants included in the final analytic sample (n = 1636). Table S3. Comparison of demographic and risk variables between participants included and excluded from the final analytic study sample. [file 1471-2350-13-14-S1.DOC]

**SUPPLEMENTARY MATERIAL**

**Supplementary Table 1.** Sensitivity analysis comparing gene-environment interaction between the entire analytic sample (n = 1636) and a dataset excluding a subsample with incomplete smoking at diagnosis data (n = 1186); all analyses adjusted for sex, age at diagnosis, age at study inclusion, family history, and year of recruitment

|  | **Ever Smoking** | | | | | | **Smoking at Diagnosis** | | | | | |
| --- | --- | --- | --- | --- | --- | --- | --- | --- | --- | --- | --- | --- |
|  | **Data from entire analytic sample (n=1636)** | | | **Data excluding subsample with incomplete smoking at diagnosis data (n=1186)** | | | **Data from entire analytic sample (n=1636)** | | | **Data excluding subsample with incomplete smoking at diagnosis data (n=1186)** | | |
|  | OR | 95% CI | p-value | OR | 95% CI | p-value | OR | 95% CI | p-value | OR | 95% CI | p-value |
| **Combined Allele Analysis** |  |  |  |  |  |  |  |  |  |  |  |  |
| Wild type at all 3 loci | 1.00 | - | - | 1.00 | - | - | 1.00 | - | - | 1.00 | - | - |
| Carrier of ≥ 1 risk allele | **0.71** | **0.56 - 0.90** | **0.005** | **0.65** | **0.48 - 0.87** | **0.003** | **0.68** | **0.51 - 0.89** | **0.005** | **0.68** | **0.50 - 0.91** | **0.009** |
|  |  |  |  |  |  |  |  |  |  |  |  |  |
| **R702W Polymorphism** |  |  |  |  |  |  |  |  |  |  |  |  |
| Wild type | 1.00 | - | - | 1.00 | - | - | 1.00 | - | - | 1.00 | - | - |
| Carrier of risk allele | 0.80 | 0.59 - 1.07 | 0.14 | 0.73 | 0.50 - 1.05 | 0.09 | 0.82 | 0.57 - 1.16 | 0.27 | 0.77 | 0.52 - 1.13 | 0.18 |
|  |  |  |  |  |  |  |  |  |  |  |  |  |
| **G908R Polymorphism** |  |  |  |  |  |  |  |  |  |  |  |  |
| Wild type | 1.00 | - | - | 1.00 | - | - | 1.00 | - | - | 1.00 | - | - |
| Carrier of risk allele | 1.00 | 0.67 - 1.53 | 0.96 | 1.05 | 0.63 - 1.77 | 0.87 | 0.93 | 0.57 - 1.49 | 0.76 | 1.01 | 0.59 - 1.69 | 0.98 |
|  |  |  |  |  |  |  |  |  |  |  |  |  |
| **1007fs Polymorphism** |  |  |  |  |  |  |  |  |  |  |  |  |
| Wild type | 1.00 | - | - | 1.00 | - | - | 1.00 | - | - | 1.00 | - | - |
| Carrier of risk allele | **0.64** | **0.49 - 0.83** | **9 × 10^-4^** | **0.57** | **0.41 - 0.78** | **5 × 10^-4^** | **0.53** | **0.39 - 0.73** | **7 × 10^-5^** | **0.55** | **0.39 - 0.76** | **4 × 10^-4^** |
|  |  |  |  |  |  |  |  |  |  |  |  |  |

**Supplementary Table 2.** Comparison of sex, age at disease diagnosis, age at study inclusion, and family history of IBD for each of the CD-associated *NOD2* risk variants among all participants included in the final analytic sample (n = 1636)

|  | **Male** | **Female** | **p-value** | **Age at Diagnosis*** | **p-value** | **Age at Inclusion*** | **p-value** | **No Family History** | **Family History** | **p-value** |
| --- | --- | --- | --- | --- | --- | --- | --- | --- | --- | --- |
| **Combined Allele Analysis** |  |  | 1.0 |  | **0.001** |  | 0.08 |  |  | 0.89 |
| Wild Type | 278 | 607 |  | 26.8 |  | 39.4 |  | 417 | 224 |  |
| Carrier of ≥1 variant | 236 | 515 |  | 25.1 |  | 38.3 |  | 366 | 201 |  |
| **R702W Polymorphism** |  |  | 0.59 |  | 0.69 |  | 0.14 |  |  | 0.007 |
| Wild Type | 418 | 898 |  | 26.1 |  | 39.1 |  | 649 | 324 |  |
| Carrier of risk allele | 96 | 224 |  | 25.8 |  | 38.0 |  | 134 | 101 |  |
| **G908R Polymorphism** |  |  | 0.07 |  | 0.35 |  | 0.35 |  |  | 0.10 |
| Wild Type | 456 | 1028 |  | 26.1 |  | 38.8 |  | 702 | 394 |  |
| Carrier of risk allele | 58 | 94 |  | 25.2 |  | 39.7 |  | 81 | 31 |  |
| **1007fs Polymorphism** |  |  | 0.74 |  | **0.001** |  | 0.17 |  |  | 1.0 |
| Wild Type | 384 | 828 |  | 26.5 |  | 39.1 |  | 572 | 312 |  |
| Carrier of risk allele | 130 | 294 |  | 24.6 |  | 38.2 |  | 211 | 113 |  |

* mean years

Adjusted α-level after Bonferroni correction for 12 tests = 0.004

**Supplementary Table 3.** Comparison of demographic and risk variables between participants included and excluded from the final analytic study sample

|  | **Included Patients** | | **Excluded Patients** | |  |
| --- | --- | --- | --- | --- | --- |
| **Characteristic** | n | % | n | % | p-value |
| **Sex** |  |  |  |  | 0.24 |
| Men | 514 | 31.4% | 230 | 29.0% |  |
| Women | 1122 | 68.6% | 564 | 71.0% |  |
| **Age at Diagnosis*** | 26.8 | ±11.1 | 24.8 | ±9.5 | **0.0004** |
| **Age at Inclusion*** | 39.6 | ±13.1 | 39.0 | ±12.1 | 0.35 |
| **Ever Smoking Status** |  |  |  |  | 0.01 |
| Ever Smoker | 947 | 57.9% | 417 | 52.5% |  |
| Never Smoker | 689 | 42.1% | 377 | 47.5% |  |
| **Smoking Status at Diagnosis** |  |  |  |  | 0.72 |
| Smoker at Diagnosis | 522 | 31.9% | 154 | 39.5% |  |
| Non-Smoker at Diagnosis | 761 | 46.5% | 236 | 60.5% |  |
| **Combined Allele Analysis** |  |  |  |  | 0.32 |
| Wild Type | 885 | 54.1% | 22 | 45.8% |  |
| Carrier of ≥1 variant | 751 | 45.9% | 26 | 54.2% |  |
| **R702W Polymorphism** |  |  |  |  | 0.54 |
| Wild Type | 1316 | 80.4% | 49 | 76.6% |  |
| Carrier of risk allele | 320 | 19.6% | 15 | 23.4% |  |
| **G908R Polymorphism** |  |  |  |  | 0.45 |
| Wild Type | 1484 | 90.7% | 70 | 87.5% |  |
| Carrier of risk allele | 152 | 9.3% | 10 | 12.5% |  |
| **1007fs Polymorphism** |  |  |  |  | 0.55 |
| Wild Type | 1212 | 74.1% | 60 | 70.6% |  |
| Carrier of risk allele | 424 | 25.9% | 25 | 29.4% |  |

* mean years

Adjusted α-level after Bonferroni correction for nine tests = 0.006
